# Research theme mapping and future directions on corruption and religion: a bibliometric analysis

**DOI:** 10.3389/fsoc.2025.1502700

**Published:** 2025-03-03

**Authors:** Salahudin, Iradhad Taqwa Sihidi, Ali Roziqin, Tinuk Dwi Cahyani, Kisman Karinda, Muhammad Firdaus, Tawakkal Baharuddin

**Affiliations:** ^1^Department of Government Studies, Universitas Muhammadiyah Malang, Malang, Indonesia; ^2^Faculty of Law, Universitas Muhammadiyah Malang, Malang, Indonesia; ^3^Department of Government Studies, Universitas Muhammadiyah Luwuk, Luwuk, Indonesia; ^4^Master Program of Sociology, Universitas Brawijaya, Malang, Indonesia; ^5^Department of Government Studies, Universitas Muhammadiyah Makassar, Makassar, Indonesia

**Keywords:** anti-corruption, corruption, religion, religiosity, liberation theology

## Abstract

Corruption harms social and economic structures whilst religion acts as a moral pillar with ethical values to fight it. Unfortunately, the relationship between the two is less explored in the literature. This present study employs a bibliometric analysis approach with data retrieved from the Scopus database. We explore the relationship between corruption and religion through the identification of trends and mapping important topics to reveal potential future research on the topic. The results show a considerable increase in the recent number of publications on the topic despite its limited literature. More importantly, the mapping illustrates that the relationship between corruption and religion has transformed focus from economic, social, and political factors to including religious variables as an important element. In particular, topics like religiosity, religious affiliation, liberation theology, education, and anti-corruption mark shifts in complexity in current discussions. Such a complexity challenges diverse perceptions of religious values and their position in linking political and economic interests. This finding implies that future research needs to adopt a holistic and contextual framework to understand the factual impact of religion on corruption. This attempt potentially expands the current thinking on the role of religion within corruption cases in shaping a more inclusive perspective that bridges anti-corruption practises in society.

## 1 Introduction

The negative impact of corruption permeates the social and economic structure of a society, posing detrimental consequences for development and sustainability (Ahmed and Anifowose, 2024; Fhima et al., 2023). Economically, corruption can harm resource allocation and hinder economic growth by destroying investor confidence and reducing public sector efficiency (Habibov et al., 2017; Kang et al., 2023). In addition, corruption exacerbates social justice through inequalities and destroys systems of fair distribution (Badur et al., 2024; Novella-García and Cloquell-Lozano, 2021). In public service, corruption can hinder people’s access to basic services such as education and health. Corruption damages moral values and integrity, undermines public trust in institutions, and forms a culture of disobedience to the law. Overall, it can be said that the effects of corruption ignite an unstable social and economic environment, impede development progress, and threaten the overall well-being of society.

Corruption may occur due to complex factors involving social, economic, political, and cultural conditions (Meričková et al., 2017; Rontos et al., 2013). At the individual level, the desire for personal gain, financial dissatisfaction, and lack of moral integrity play key roles (Han, 2023; Maciel and de Sousa, 2018; Manase Kudzai Chiweshe, 2021). At the structural level, weak legal systems, lack of government transparency, and weak supervision are amongst the driving factors of corruption (Marcela Porporato, 2016; Meiryani et al., 2021; Montes and Luna, 2021). Unclear regulations and wealth inequality can also spark corrupt practises (Dusha, 2019; Tawiah et al., 2024). Additionally, political factors such as nepotism and patronage also initiate a culture of corruption in a country (Loring, 2022; Singh, 2005). Therefore, attempts to prevent and resolve corruption cases call for collective improvements in the intertwining aspects ranging from individual integrity to institutional reform to strengthen the legal system.

Discussions on corruption and religion are closely linked to ethics, morality, and the values that underlie human behaviour (de Oliveira Leite et al., 2021; Manase Kudzai Chiweshe, 2021). Religion acts as a moral guide for many individuals, establishing the principles of integrity, honesty, and social responsibility. Interestingly, complex dynamics between religion and corruption are largely documented in the literature (Maswandi et al., 2022; Sommer et al., 2013; Zelekha and Avnimelech, 2023). This is because all religious teachings condemn corrupt practises, but leaders involved in the corrupt practises likely claim to adhere to a particular religion (Karim et al., 2023; Wijaya, 2014; Xu et al., 2017). This phenomenon indicates the urgent need for in-depth study into the relationship between corruption and religion, which includes moral aspects, and a careful understanding of the complex interactions between religious factors and corrupt behaviour in society.

Although research on corruption has involved various scientific disciplines and social aspects, special attention to the specific correlation between corruption and religion is limited. Much research has explored the root causes and impacts of corruption from legal, economic, and sociological perspectives, yet the factor of religion remains underexplored. There is a dearth in the literature that specifically uncovers the complex dynamics between religious values, morality, and corrupt practises. This is a potential research gap for future studies that explore the role of religion in the prevention and resolution towards corruption, specifically on how religious values may shape ethical behaviour in social and institutional environments. A more thorough understanding on these variables will contribute significantly to the efforts to eradicate corruption with a more holistic approach.

Hence, this present study aims to explore and identify trends, patterns, and contributions of academic literature on corruption and religion using a bibliometric analysis approach. Such an approach can compile and analyse bibliographic data relevant to the topic, including scientific articles, books, and other publications. By detailing research developments over time, this present study attempts to provide a comprehensive picture of the knowledge evolution related to corruption and religion. The results of this present study can identify central topics, main concepts, and potential research gaps in the relationship between corruption and religion. This research seeks to make an important contribution to understanding the relationship between the two, which paves the way for further research that unravels the dynamics between corruption and religion.

## 2 Literature review

### 2.1 Corruption: a multidisciplinary perspective

From a legal perspective, corruption refers to the abuse of power or position to obtain personal gain or group interests. Abundant legal studies have explored the root causes of corruption, including weak legal systems, law enforcement, and lack of transparency in regulations (Gutterman, 2023; Søreide, 2016). The literary discussion has been centred around the effectiveness of the legal system in handling corruption cases, along with the challenges and weaknesses that may arise during the eradication efforts. The economic perspective, on the other hand, views corruption as a factor that harms resource allocation and hinders economic growth. This view mainly focuses on the impact of corruption on the public and private sectors through evaluations on budget allocation, investment, and investor confidence. Many studies attempt to measure the direct and indirect economic costs of corruption, which include the fall of market trust and potential long-term investment losses (Aïssaoui and Fabian, 2022; Habibov et al., 2017; Kang et al., 2023; Shi and Pan, 2018).

From the sociological perspective, corruption is understood as the result of social and cultural factors (Zaloznaya and Gerber, 2021). This perspective mostly identifies the practises of cultural norms, nepotism practises, and patronage, which can strengthen or undermine anti-corruption systems. Sociology scholars often highlight the effect of corruption on social structures, inequality, and power dynamics in society (Heath et al., 2016; Moya Díaz and Paillama Raimán, 2022). Studies on corruption through the lens of Sociology have revealed how social and cultural factors can contribute to the persistence of corruption at various levels in society. Meanwhile, from a political perspective, corruption is strongly linked to nepotism and patronage practises. This perspective often analyses how policies and decisions can be influenced by personal relationships and undue loyalties, leading to the granting of privileges and advantages to certain individuals or groups (Jha, 2023; Keegan, 2024; Oluseye, 2024), which can be equated to corrupt practises.

Last, the psychological perspective views corruption from individual motivation and ethics. In-depth psychological research shows that psychological factors can drive individuals to engage in corrupt acts, such as the need for personal gain, financial security, or distorted ethical justifications (Abraham et al., 2018; Zaloznaya, 2014). Studies on corruption through the lens of psychological perspective denote the pivotal role of psychological factors within social and economic conditions, which eventually lead to corrupt behaviour. It is noteworthy that the complexity of individual motivations is behind acts of corruption (Modesto and Pilati, 2020). Meanwhile, the global perspective regards corruption as an internal problem of a country, which poses a broad impact on relations between countries and at the international organisational level. Global studies on corruption primarily examine the effect of corrupt practises on economic cooperation, diplomacy, and foreign policy between countries (Hafner-Burton and Schneider, 2019; Haw et al., 2023; Radu et al., 2015).

### 2.2 Corruption: ethics and morality from the religion perspectives

Religion is a moral pillar that affirms principles such as honesty, integrity, and social responsibility (de Oliveira Leite et al., 2021; Manase Kudzai Chiweshe, 2021). This present study focuses on how religious teachings influence individual views and behaviour towards acts of corruption. This objective promotes a broader understanding on the complex relationship between corruption and the ethical values championed by the world’s major religions. In the Islamic context, studying corruption from the perspective of ethics and morality plays a central role in shaping the behaviour and actions of Muslims. Islamic teachings emphasise ethical values, such as honesty and integrity, and condemn all forms of corruption (Alazzabi et al., 2020; Rahbarqazi and Mahmoudoghli, 2020). Besides, the concept of amar ma’ruf nahi munkar (commanding what is good and preventing what is bad) ideally strengthens the responsibility of Muslims in fighting corruption (Setia et al., 2023).

Not only in Islam, ethical and moral values opposing corruption have also beco-me a strong foundation in other religions throughout the world. In Christian teachings, the concepts of integrity, honesty, and justice also play a key role in condemning acts of corruption (Kidd, 2024; Lang, 2014; Kidd, 2023). The Bible teaches the important value of living under moral principles that promote common prosperity and social justice (Lang, 2014). Likewise, Hinduism and Buddhism teach ethical values that emphasise the importance of honesty, good actions, and justice in everyday life (Fisher, 2015; White, 2006). Therefore, literature analysis that covers a variety of religious teachings can provide a deeper understanding of universal ethical values that stretch across various beliefs, whilst explaining how these values become a common foundation in dealing with the problem of corruption.

It thus can be seen that religious teachings, including Islam, Christianity, Hinduism, Buddhism, and most likely others, consistently uphold ethical values such as honesty, integrity, and justice, whilst condemning all forms of corruption. Challenges in the integration of religious ethical values as a moral pillar to combat corruption primarily lie in the differences in religious interpretation and practise in a heterogeneous society (Anteby-Yemini, 2023; Etemadinia and Jahromi, 2022; Othman, 2023). Additionally, the identification of effective strategies to transform religious values into real actions in diverse social and political contexts has also been challenging (Gokcekus and Ekici, 2020). Consequently, there must be a collaborative effort between religious leaders, civil society, and the government to develop an inclusive and sustainable approach, as well as build awareness on ethical values to fight corruption (Chaney and Sahoo, 2020).

Overall, a deep understanding of the universal ethical values held by religions can be a common basis for combating corruption. This calls for collaboration across religious and societal sectors to formulate more effective strategies for translating religious values into actions in social and political contexts. Moreover, joint efforts between religious leaders, civil society, and government potentially form a strong front in the fight against corruption, ensuring that honesty, integrity, and justice are the main moral pillars that guide individual behaviour and decisions. Thus, the ethical values championed by the world’s major religions can provide a strong foundation for building a clean, just, and socially responsible society.

## 3 Methods

**Figure 1:**
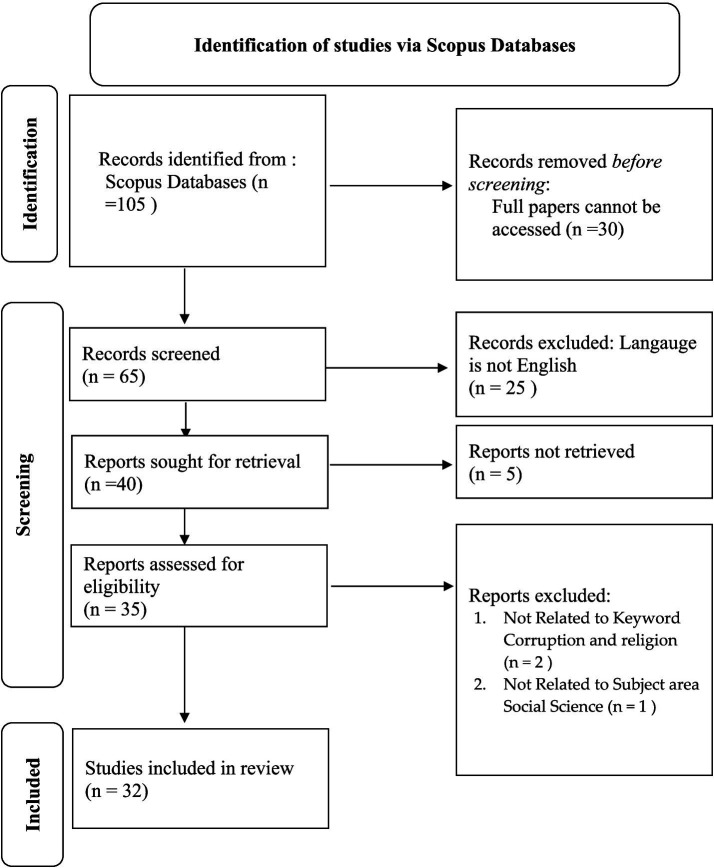
Stages of document retrieval.

This study employs a bibliometric analysis approach retrieved from the Scopus database as the main source. We decided to not limit data collection based on year of publication, subject area, affiliation, or document type to gain a comprehensive view towards the development on studies related to corruption and religion in the academic literature. By removing restrictions, we expect to capture trends and changes over time and accommodate subject diversity. The choice of keywords, which are “Corruption” and “Religion,” is designed to provide a clear focus on those two mentioned aspects. The results of data collection carried out on 14 January 2024 listed 32 published documents, whose stages were carried out in accordance with the PRISMA (Preferred Reporting Items of Systematic reviews and Meta-Analyses) protocol, namely identification, screening, and including articles from the Scopus database (Figure 1).

There are two important criteria in data retrieval from the Scopus database, namely inclusion and exclusion criteria. Inclusion criteria are the criteria chosen as the focus of this study, namely document by year and document by author. Both criteria are included in the reference retrieval stage in Scopus, namely at the initial stage of entering the keyword “religion and corruption” in the search column in the Scopus reference search column, then document filtering or document restrictions are carried out only on the year and author criteria. Based on these criteria, we took graphic data in the form of publication trends from year to year and data on the names of authors on the topic of religion and corruption. Other data taken were RIS and CSV data, namely data used for topic and theme mapping analysis with Vosviewer. Whilst the exclusion criteria are the criteria used to limit references outside of documents by year and documents by author. These criteria include subject area, country, keywords, and other criteria listed in the Scopus database.

Following the results of data collection, the bibliometric analysis continues by utilising the analysis tools on the Scopus database page to visualise the data. The visualisation includes the number of documents by year of publication, subject area, affiliation, and number of citations, providing a comprehensive picture of the trends and impact of the research. To maximise the analysis, we used VOSviewers to detail the mapping of influential research topics related to corruption and religion. This step allows the identification of relationships and research patterns that emerged from a broad data set. Finally, both visualisation results from Scopus and VOSviewers are presented in tables and figures (see Section 4), providing a deep understanding of the dynamics of academic literature related to corruption and religion.

Validation and reliability are indeed critical in this present study to ensure the accuracy and reliability of the results. Validation was carried out by ensuring that the data collection and bibliometric analysis methods used comply with established standards and procedures. In this case, the re-verification of Scopus-indexed documents is carried out to ensure the validity of the data source. Meanwhile, reliability is obtained through the manual operation of analysis tools, which was VOSviewers, where the results can be reproduced in a similar way to retest the credibility of the findings. The clear selection of keywords at the initial stage, namely “Corruption” and “Religion” also supports the reliability of the bibliometric analysis by providing a consistent framework for identifying related literature. By so doing, this present study can make a significant contribution to the understanding of the relationship between corruption and religion in academic literature.

## 4 Results and discussion

### 4.1 Corruption and religion: analysis of global research trend

This section investigates the global research trends regarding corruption and religion, focusing on the number of documents collected from the Scopus database, which includes year of publication, subject area, affiliation, and number of citations. Taking these variables into account, we identified research trends over time, detected dominant study areas, revealed the contribution of certain affiliates, and assessed the impact of the research through the number of citations. This present study does not only detail quantitative dimensions, but also provides a comprehensive picture of the extent to which this conversation has emerged in the global scientific arena.

**Figure 2:**
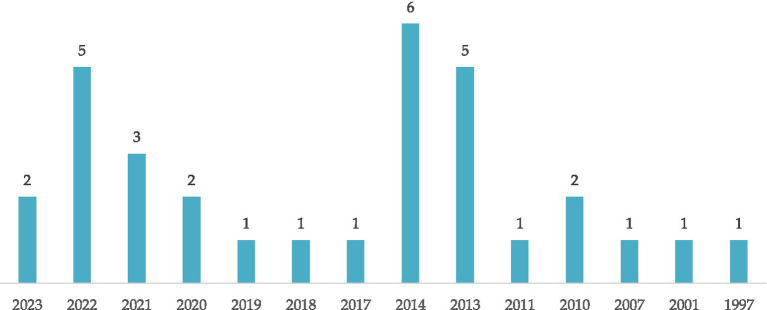
Number of documents about corruption and religion based on the year of publication.

Figure 2 depicts the distribution of the number of documents about corruption and religion based on the year of publication. It can be seen from Figure 2 that research on corruption and religion has varied over time. The peak number of documents occurred in 2014 with six publications, indicating high potential for conversation and research in that period. Although there is a slight decrease in subsequent years, especially in 2022 and 2023, the number of documents remains relatively significant. A slight decline is apparent in 2020 and 2021, but still showing a continued interest in this topic. The number of publications tends to be lower in the previous years with only one to two documents annually. This finding reflects dynamic developments in research interest in this domain whilst showing the potential for increased focus in certain periods.

However, the presented data shows that discussions on corruption and religion have not gained popularity in academic literature, especially in the years with a limited number of publications. Despite the increasing trend over time, the time when publication frequency is low highlights the need for continued exploration of these topics. The relationship between corruption and religion should receive more attention as a central aspect in the social and ethical development of society. This potential gap emphasises the need for future research that understands more deeply the complexity, and impact of the interaction between these two variables. A continued exploration can produce new insights, expose unresolved debates, and enrich an understanding of the role of religion in preventing and overcoming corruption in various social and cultural contexts. Based on this lack of popularity, the complexity of corruption and religion requires further studies that investigate the link between the two.

Table 1 provides an overview of the document distribution related to corruption and religion based on the subject area. The data shows that the subject area is widely spread across various scientific disciplines. It is readily apparent from the table that Social Sciences dominates with 17 documents, indicating significant interest from this perspective in the complexities of interactions between corruption and religion in society. Meanwhile, Economics, Econometrics, and Finance followed with 15 documents, reflecting high interest from an economic perspective in the impact and strategies for preventing corruption by considering religious values. Arts and Humanities also contribute significantly with 11 documents, showing an understanding of the ethical and moral dimensions of the relationship between corruption and religion. Interestingly, closely related disciplines with corruption like Business, Management and Accounting; and also Agricultural and Biological Sciences, Environmental Science, and Multidisciplinary, each contributed a lower number of documents that show the diversity of approaches and research focus. It can be concluded that the complex relationship between corruption and religion has been studied diversely across scientific disciplines.

**Table 1:** Number of documents about corruption and religion by subject area.

Subject area	Documents
Social Sciences	17
Economics, Econometrics and Finance	15
Arts and Humanities	11
Business, Management and Accounting	7
Agricultural and Biological Sciences	1
Environmental Science	1
Multidisciplinary	1

**Figure 3:**
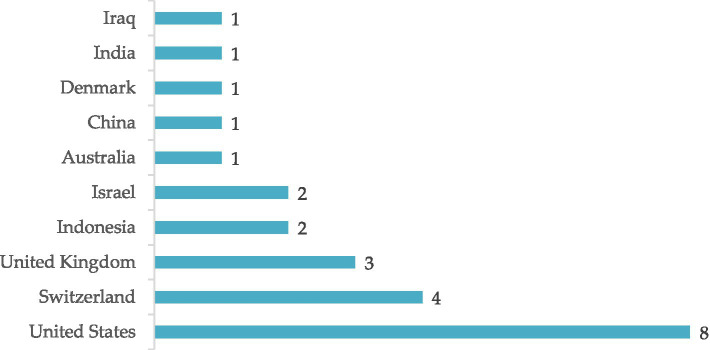
Number of documents on corruption and religion based on author affiliations.

Figure 3 illustrates the number of documents on corruption and religion based on author affiliation, with a focus on the country or region of domicile. The data reflect the global distribution of this research from countries across continents. The United States is the largest contributor with eight documents, indicating the high interest and active contribution of researchers in that country to this topic. Switzerland and the United Kingdom contributed four and three documents respectively, indicating the significant involvement of European researchers in discussing the links between corruption and religion. Indonesia and Israel each contribute with two documents, showing that this topic attracts interest and participation from researchers in Asian and Middle Eastern countries. Last, countries such as Australia, China, Denmark, India, and Iraq each contributed one document, adding a broader international dimension to the topic.

Overall, tracing global research trends on corruption and religion reveals significant interest and contributions from various parts of the world. Despite the periods of low publication frequency, the continued increase in the number of published documents indicates that this topic is increasingly recognised and receiving attention in the academic literature. The research focus involves various scientific disciplines, especially in the fields of Social Sciences, Economics, and Arts and Humanities, reflecting the complexity and multidimensionality of the phenomena of corruption and religion. Meanwhile, variables such as author affiliation highlight the important role of researchers from different countries in contributing to this global discussion. All these findings imply the need to increase global conversation regarding the link between corruption and religion, as well as revealing the need for further research that explores the specific aspects that have not yet been fully addressed.

In addition, this present study details research documents in their contribution to discussing the relationship between corruption and religion based on the number of citations. This section only presents the five most-cited documents over the years.

Table 2 presents information regarding the most-cited documents on corruption and religion. Analysis of these documents is the key to identifying works with significant impact on academic literature. The most-cited documents show the works’ important role in shaping and enriching the understanding of the relationship between corruption and religion. In general, the most-cited documents become the indicators of influence and research relevance in the scientific community (Aksnes et al., 2019). A high citation index indicates the significant contributions of the research itself due to the peer recognition and adoption by other research in various contexts (Baharuddin et al., 2022; Malik et al., 2023; Tahamtan and Bornmann, 2019). The citation index reinforces the value of the research as an influential reference source whilst increasing a researcher’s visibility and reputation in a particular field. Thus, an analysis of the most-cited documents on corruption and religion not only provides information on the work’s impact, but also shows the extent to which this research contributes to the development of general thinking and understanding.

**Table 2:** The most-cited documents on corruption and religion.

Title	Year	Citation
Corruption and religion adding to the economic model	2001	159
Religion, corruption, and the rule of law	2013	50
An Analysis of the effect of culture and religion on perceived corruption in a global context	2014	45
Does religion matter to corruption? Evidence from China	2017	40
Does faith limit immorality? The politics of religion and corruption	2013	22

Looking at the table, the first listed document produces significant findings on the relationship between corruption and economic factors whilst incorporating the role of religion, especially in the context of Christianity. Interestingly, the findings show that economic factors, which are real income per capita and inflation rates, have a greater impact on corruption levels than religious factors. This research identified that within Christianity, particularly amongst Protestants and Anglicans, lower levels of corruption are documented (Paldam, 2001). This research also provides an in-depth understanding of the complexity of the relationship between corruption, economic factors, and religion, showing that these dimensions are intertwined in shaping corruption patterns in various countries. The main contribution of this paper lies in the application of the economic-cultural model to explain global corruption patterns, revealing that religious factors (in this case, Christian) play a pivotal role in shaping the characteristics of corruption at the international level despite the dominant impacts of the economic factors. These findings provide new insights into the corruption and religion literature, which has become the basis for further debate and policy regarding preventing and overcoming corruption in economic and religious contexts.

The second document listed in the table covers the association between the rule of law, corruption, and a country’s religious heritage. With a sample of 207 countries, this study reveals that the rule of law and levels of corruption are closely related to the country’s religious heritage. This partly explains the relationship between religion and economic growth that has been largely documented in the literature. Interestingly, this study denotes that their findings changed when some variables were controlled, despite the change being caused by differences in sample composition and not by the effects of the control variables themselves (North et al., 2013). This research mainly contributes to the confirmation of distinct characteristics between the effects of additional control variables and the sample composition effects produced through cross-country analyses. By carefully identifying the links between the rule of law, corruption, and religious aspects, this research successfully provides a deeper understanding of the complexity of these relationships. The findings of this research imply a new perspective in the literature on corruption and religion, highlighting the mediating role of law in linking religious aspects with the level of corruption and economic growth of a country.

The third document investigates the impact of culture and religion on perceptions of corruption on a global scale during the 2000–2010 period. The results show that cultural and religious differences significantly correlate with levels of perceived corruption, even after controlling the economic and political factors. People of Non-Protestant Christianity, Islam, and Other/No Religion tend to be positively associated with corruption, whilst Buddhism and Hinduism do not differ significantly from Protestant Christianity. From a cultural perspective, European cultural groups (other than Anglo-Saxon) tend to be associated with corruption, but this effect can be offset by more effective political governance. Meanwhile, non-European cultural groups tend to be associated with higher levels of corruption although the impact may be mitigated by higher perceptions of political legitimacy or political effectiveness (Mensah, 2014). These findings provide further understanding of the complexity of the relationship between religion, culture, and corruption, and provide a basis for more contextual corruption prevention policies at the global level.

The fourth document aims to understand the impact of religion on corruption, especially in the Chinese context. It uses provincial-level panel data from 1998 to 2009. Empirical results show that bureaucratic corruption does not correlate with local religious heritage, indicating that religious culture plays a positive role in curbing official corruption. It thus can be said that religion influences political preferences and work ethics. These findings also show that the negative relationship between religion and corruption is weaker in provinces with stronger law enforcement, identifying a substitution effect between religious ethics and legal oversight in curbing corruption. This research also found that the anti-corruption effect of China’s indigenous religions, namely Taoism and Buddhism, is more significant than that of foreign religions, such as Christianity and Islam (Xu et al., 2017). These findings offer new insights into the literature on corruption and religion with its evidence on the relationship between religion and corruption, especially in the Chinese context where previous studies and data sources are limited.

The last document listed in the table critically evaluates the relationship between religiosity and ethical behaviour. This research argues that religion is systematically related to levels of corruption, which is contingent on the existence of democratic institutions. In democracies, where political institutions are designed to discourage corrupt behaviour, the morality imparted by religion is associated with reduced levels of corruption. On the other hand, in systems without democratic institutions, moral behaviour does not always mean avoiding corrupt methods (Sommer et al., 2013). It thus can be concluded that in non-democratic contexts, religion is not associated with reduced corruption. The data analysis involving 129 countries for over 12 years in this study employs the individual-level analysis of World Values Surveys data, which provides strong support to the existing theory. The correlation between religion and reduced corruption is conditional based on the extent to which political institutions are democratic. This research contributes significantly to the literature on corruption and religion by illustrating that the relationship between the two depends on the context of political democratisation.

Taken together, these five documents provide deep insights into the complexities of the relationship between corruption and religion. The first document highlights the dominance of economic factors in influencing levels of corruption, pointing out that religion, especially in the Christian tradition, provides a significant additional explanation. The second document emphasises the role of law and religious heritage that influences a country’s level of corruption. Meanwhile, the third document illuminates the role of culture and religion in the global perceptions of corruption, showing that cultural and religious differences remain correlated with levels of corruption even after controlling for economic and political factors. The fourth document explores the impact of religion on corruption in the Chinese context, revealing the positive role of religious culture in curbing corruption, particularly in China’s indigenous religions. Finally, the fifth document empirically establishes the relationship between religion, ethics, and levels of corruption, showing that this relationship depends on the context of political democratisation. Altogether, findings from these studies conclude that the relationship between corruption and religion is complex, and influenced by economic, legal, cultural, and political context factors.

### 4.2 Corruption and religion: mapping the research topics

This mapping aims to identify the dominant research focus and explore the contribution of various scientific disciplines in understanding the complexity of the relationship between corruption and religion. This section provides a comprehensive overview of the variety of perspectives applied by researchers in detailing key aspects of the interaction between corruption and religion. Thus, mapping research topics can enrich insight into academic discussions on the relationship between corruption and religion. Figure 3 below displays the mapping of research topics that influence discussions about corruption and religion. Although a variety of dominant topics have contributed significantly to the understanding of the complex relationship between corruption and religion, several recent topics stand out and require continued exploration. The focus on religiosity, religious affiliation, liberation theology, education, and anti-corruption indicates a shift and complexity in the current discussion.

**Figure 4:**
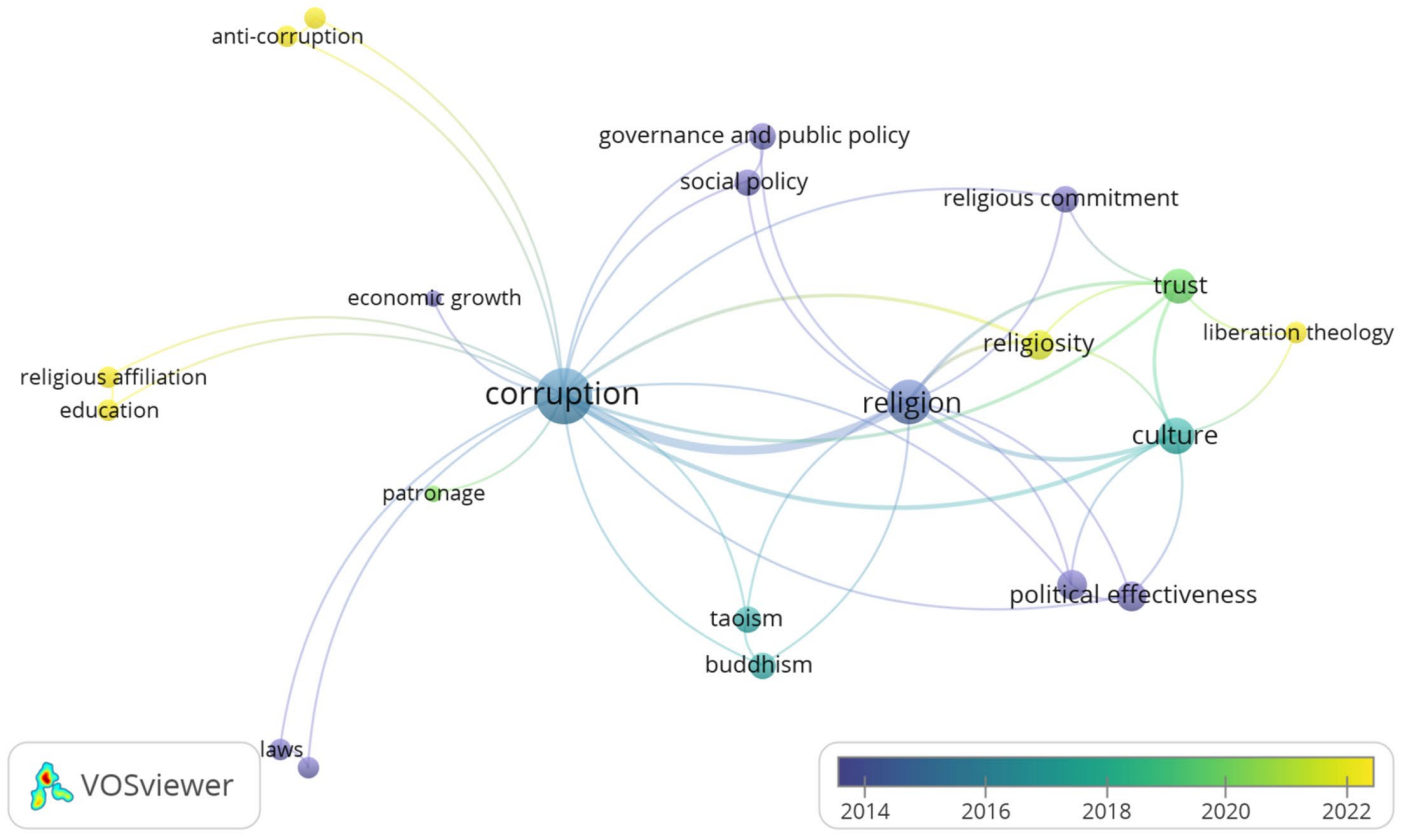
Topics that influence discussions on corruption and religion.

Religiosity, as an important dimension in understanding the relationship between religion and behaviour, highlights the complexity of interaction between an individual’s or community’s religious level and corrupt behaviour. Religiosity encompasses the spiritual, ritual, and moral dimensions of a person’s life, which potentially form the basis of ethics and values that may influence corruption-related decisions (Gokcekus and Ekici, 2020; Sommer et al., 2013). Individuals or communities with high levels of religiousness tend to have stronger moral commitments, which can limit the possibility of engaging in corrupt actions. Meanwhile, low religious levels reflect a lack of moral orientation that opens doors to corrupt behaviour (Frisdiantara et al., 2017). Therefore, further understanding on the influence of the religious level on attitudes and behaviour regarding corruption can provide valuable insights into detailing the role of religion in the formation of social integrity and ethics in society (Figure 4).

Next, religious affiliation has also become the focus of research on the exploration of perceptions and actions of corruption. Religious affiliation refers to an individual’s or group’s identification with a particular religious denomination that can shape the worldview, values, and social norms that underlie their everyday behaviour (Gokcekus and Ekici, 2020; LeClair, 2019). The dimension of religious affiliation in the context of corruption tries to understand whether members of a particular religious community tend to have different attitudes or actions regarding corruption compared to other religious groups or those without religious affiliation. Through this understanding, a deeper insight is gained into the role of religion as a determining factor in shaping integrity and morality in social actions, including how perceptions of corruption are reflected and overcome in various religious groups.

A deeper understanding of liberation theology, as the next aspect, widens the possibility of understanding the relationship between theological understanding and resistance to corruption. Liberation theology is rooted in Latin American theological contexts, which combines the principles of social justice and liberation from inequality with religious principles (Portilla, 2022). In the context of corruption, this understanding can provide a unique perspective on how religious teachings can empower individuals and communities to fight and overcome corruption. In addition, an understanding of liberation theology can also reveal ways in which religion can be a source of inspiration for social action to establish a just society free from corrupt practises. Such exploration between liberation theology and corruption can also recognise the role of religion as a transformational force to fight injustice and inequality at various social levels.

Education is the next aspect involved in the quest for understanding the link between corruption and religion. It acts as a tool for transmitting religious moral and ethical values, and plays an important role in forming individual awareness towards the negative consequences of corrupt behaviour (Kumar and Sharma, 2023; Mohammed et al., 2023). Through education, society can be equipped with a deeper understanding on the principles of justice, transparency, and integrity that underlie religious teachings. Besides, education can be a means of developing a critical attitude towards corruption whilst encouraging preventive action (Suyadi et al., 2020). Education that focuses on religious values can shape character and morality, and encourage individuals to become agents of positive change in preventing and overcoming corruption. Therefore, the integration of religious values in the education curriculum can make a significant contribution to create a more moral society that is capable of curbing corruption.

Last, anti-corruption strategies are often influenced and enriched by religious values to create a clean and just society. Anti-corruption movements powered by religious principles can provide a strong moral foundation for mobilising community support and participation (Al-Maeeni et al., 2022; Karim et al., 2023). The concepts of justice, honesty, and accountability rooted in religious teachings have often become the basis for formulating anti-corruption policies. In addition, religious values can form social norms that condemn corrupt behaviour, and strengthen individual moral responsibility towards society (Xu et al., 2017). The integration of religious values in anti-corruption campaigns will provide a deeper ethical dimension, creating collective awareness of the dangers of corruption. This will encourage society’s active participation in efforts to prevent and overcome corruption. It can be said that religious involvement in the anti-corruption movement will create both a moral stage and a driving force to shape anti-corruption behaviour at all levels of society.

Several research results show that religion and corruption are two conflicting terms, where religion teaches morals and goodness, whilst corruption is a bad practise of humans in life in the world. However, religion has a moral obligation to ensure that humanity must avoid corrupt practises in their lives wherever humans are (Brandtstädter, 2013; Sullins, 2003; Sumaktoyo and Muhtadi, 2022). Religion plays an important role in teaching humanity not to cheat, steal, take other people’s rights, use power and authority for personal or group interests, on the contrary humanity is obliged to act fairly, defend the truth, use power and authority for the benefit of humanity, make policies that favour the public interest, ensure equality and welfare are felt by all humanity (Al-Maeeni et al., 2022). The role of religion does not have to be institutionalised in a government/state policy, but this role can be carried out through religious pulpits which are a public means of conveying religious teachings (Gouda and Park, 2015).

However, what is much more important is that religious values can be applied in the lives of humanity wherever humanity plays a role. Given the importance of the role of religion in eradicating corruption, all religions need to build synergy and collaboration between each other who take roles in their respective areas of attention (Singh, 2016). In principle, all religions have similarities in viewing corrupt practises as a form of sin that has a negative impact on those who do it and on all of humanity. This synergy can be done by forming a special religious institution that plays a role in providing education, socialisation, advocacy, and awareness to the people to participate in anti-corruption activities or conduct massive anti-corruption campaigns (Wijaya, 2014). In addition, religions can also collaborate in designing anti-corruption education curricula that can be applied to formal and non-formal schools at every level. Religious figures need to act as role models for the anti-corruption movement which is expected to have a positive influence on the birth of cadres and anti-corruption generations who play a role in public institutions, both as public officials, politicians, activists, and others. Religious schools also need to build synergy in ensuring that their respective graduates side with the anti-corruption movement (Marshall, 2008).

Religious institutions also need to build collaboration and synergy with state institutions in anti-corruption activities. Religious institutions can provide a theological basis for state institutions in formulating policies, programmes, and anti-corruption activities so that the public can understand that corrupt practises are crimes that are prohibited by religion and have a negative impact on the future of humans both in this world and in the hereafter (Nicholson et al., 2009). State institutions need to provide space for religious institutions/religious figures to take part in anti-corruption activities, for example inviting religious figures in policy formulation activities and implementing anti-corruption programmes and activities at all levels of government institutions. Through collaboration between religious institutions and state institutions, corrupt practises can at least be controlled properly so that state resources can be maximised for the benefit of the people/public, nation and state (Singh, 2015).

Considering the important role of religion, it is important to conduct studies on religion and corruption continuously so that a scientific concept is presented that comprehensively explains the role of religion in anti-corruption activities. In conclusion, the mapping of research topics around corruption and religion shows substantial diversity in attempts to understand the complex interactions between religious, cultural, and economic factors. There are various dominant topics such as religiosity, religious affiliation, liberation theology, education, and anti-corruption, which each holds a critical role in shaping discussions and research in this field. In detail, religiosity provides insight into the complexity of the interaction between individual and collective religious levels and corrupt behaviour, whilst religious affiliation provides information on its impacts towards perceptions and actions of corruption. Next, liberation theology promotes further exploration on the relationship between theological understanding and resistance to corruption. Education can shape awareness of the negative impacts of corruption whilst the development of anti-corruption strategies influenced by religious values will create a moral foundation for community participation to prevent and overcome corruption. Knowledge-based understanding of these topics makes a valuable contribution to policy development, and corruption prevention, and ignites broader social change.

### 4.3 Corruption and religion: a future direction

Studies on corruption and religion have provided valuable insights despite their limitations and calls for further research related to their connection with contemporary social and economic dynamics. Several research topics, such as religiosity, religious affiliation, liberation theology, education, and anti-corruption, show the potential for developing more in-depth and contextual future studies. Through future research, the complexity of the relationship between religious values, acts of corruption, and efforts to prevent corruption in various contexts can be further explored.

Besides, the trend of findings as explicated in this present study shows that discussions on the values of Islamic teachings are limited, leaving a literature gap in the research that explores the role of Islam in preventing corruption. We suggest further studies that focus more on an in-depth understanding of the roles of Islamic ethical principles in shaping attitudes towards corruption, and investigating the contribution of Muslim communities in anti-corruption movements. The studies can also be about the implementation of justice and honesty values in social and economic systems inspired by Islamic teachings. Further exploration on these dimensions can enrich the current insights into the role of Islam in supporting integrity and social justice, whilst providing a valuable basis for contextual policies that address corruption. Religious leaders such as *Mufti,* and religious educational institutions such as Islamic boarding schools, can be an important element in bridging the understanding of Islamic values with anti-corruption practises in society.

Further studies are also expected to enrich understanding on the relationship between religion and corruption by adopting a comparative perspective from different religious traditions. Research involving diverse religions can provide holistic insights into differences in anti-corruption views, values, and practises amongst different religious communities. Through comparative studies, further studies potentially explore the influences of teachings, values, and culture in certain religions towards attitudes and actions towards corruption. A comparative study provides opportunities to understand the contextual factors that moderate the influence of religion in an increasingly connected global context. The results of such studies will imply a deeper understanding of the complexity of the relationship between religion and corruption, enabling the adoption of more contextual and effective prevention and response strategies in various religious contexts.

Understanding the complex relationship between religion and corruption is an urgent need to curb corruption cases in the future. First and foremost, religion still becomes a moral compass and ethical pillar in society, hence unravelling the ways in which religious values influence attitudes towards corruption may shape more moral character and behaviour in society. Second, the rapid growth of social, economic, and political complexity reflects the need for a stable stance to guide actions. Religion, in this case, can act as a moral guide in the face of the increasingly sophisticated and disturbing challenges of corruption. Tapping into the ways religion may prevent corruption can contribute to the efforts in formulating strategies for curbing corruption cases in line with religious values and norms.

In this era of globalisation and increasing connectivity, understanding the fundamental role of religion in preventing corruption can provide broader and more contextual insight. It is important to develop solutions that can be applied globally whilst taking into account the diversity of religious values around the world. In many countries, religion is still the force for mobilisation and social change. The influence of religious leaders and religious institutions in confronting corruption can be the key to shaping public opinion, creating social norms, and encouraging policy change. Thus, understanding the relationship between religion and corruption in the future lies in the ability to form a more moral society, face the challenge of corruption with a values-based approach, and create solutions that can apply globally in the context of religious diversity.

Despite its urgency, several challenges are prevalent in the quest to understand the relationship between corruption and religion. Amongst the primary challenges is the complexity and diversity of interpretations of religious values in the communities. Each religion generally has various interpretations, and understanding of the values within them can vary significantly (Anteby-Yemini, 2023; Etemadinia and Jahromi, 2022; Othman, 2023). In addition, the risk of misusing religion for political or economic interests can also cloud relationships that should be purely ethical (Giorgi, 2022; Marchetti et al., 2022). The changing social and political contexts and complex global dynamics also add to the level of difficulty in identifying the real impact of religion on corrupt behaviour. It is noteworthy that future studies must pay attention to the context and diversity of interpretations of religious values, whilst considering changes in social dynamics to obtain a more holistic and accurate understanding.

## 5 Conclusion

Global research on corruption and religion reflects significant interest and contributions from various disciplines around the world. Although periods of low publication frequency have occurred, the increase in the number of documents in recent years indicates that this topic is increasingly recognised and becoming a focus in the academic literature. Our mapping research topics found that the understanding of the relationship between religion and corruption has undergone an evolution, from a focus on economic, social and political factors, to the inclusion of religious variables as an important element. Research topics such as religiosity, religious affiliation, liberation theology, education, and anti-corruption mark shifts and complexity in current discussions. The role of religion in anti-corruption activities can be carried out in the form of education, socialisation, and anti-corruption campaigns through religious pulpits, both delivered verbally and through actions such as advocacy, supervision, and control of state apparatus. In addition, collaboration and synergy between religious institutions and state institutions need to be strengthened for the agenda of eradicating corruption.

However, the challenges of the complexity and diversity of interpretations of religious values in various communities, along with the risk of instrumentalisation of religion for political and economic interests, must be taken into account. In this context, future research needs to consider a holistic approach and contextual framework to understand the visible impact of religion on corrupt behaviour. Future studies can also make further contributions by deepening understanding of the role of Islam in the context of corruption, which is still minimal in this domain. This potentially initiates a diversity of perspectives, which bridge Islamic values towards anti-corruption practises in society.

To conclude, a more thorough understanding on the relationship between religion and corruption is urgently needed to shape a more moral society and face the challenge of corruption with a values-based approach. Within the current globalisation, the inclusion of religious values into the studies of corruption also enables the development of solutions that can be applied globally, as well as taking into account the diversity of religious values around the world. This present study provides a strong basis for guiding further research in efforts to understand, prevent, and overcome corruption through the lens of religious values.

## Data Availability

The original contributions presented in the study are included in the article/supplementary material, further inquiries can be directed to the corresponding author.

## References

[ref1] Abraham J. Suleeman J. Takwin B. (2018). The psychology of corruption: The role of the counterfeit self, entity self-theory, and outcome-based ethical mindset. J. Psychol. Educ. Res. 26, 7–32.

[ref2] Ahmed A. Anifowose M. (2024). Corruption, corporate governance, and sustainable development goals in Africa. Corporate Govern. Int. J. Bus. Soc. 24, 119–138. doi: 10.1108/CG-07-2022-0311

[ref3] Aïssaoui R. Fabian F. (2022). Globalization, economic development, and corruption: A cross-lagged contingency perspective. J. Int. Bus. Policy 5, 1–28. doi: 10.1057/s42214-020-00091-5

[ref4] Aksnes D. W. Langfeldt L. Wouters P. (2019). Citations, Citation Indicators, and Research Quality: An Overview of Basic Concepts and Theories. SAGE Open 9, 1–17. doi: 10.1177/2158244019829575, PMID: 34290901

[ref5] Al-Maeeni M. K. A. Al-Juhaishi H. M. A. Aljburi K. H. S. Shaker R. M. Alghazali T. Mohameed D. A. A.-H. . (2022). Role of Religion and Traditions in Prevention of Corruption. Croatian Int. Rel. Rev. 28, 58–79.

[ref6] Alazzabi W. Y. E. Mustafa H. Abdul Latiff A. R. (2020). Corruption and control from the perspective of Islam. J. Financial Crime 27, 355–368. doi: 10.1108/JFC-02-2019-0020

[ref7] Anteby-Yemini L. (2023). Negotiating Gender and Religion: Comparative Perspectives from Judaism and Islam. J. Fem. Stud. Relig. 39, 187–205. doi: 10.2979/JFS.2023.A908318

[ref8] Badur M. M. Yılmaz E. Sensoy F. (2024). Do corruption and inequality shape sustainable development? Evidence from the post-soviet countries. Int. J. Soc. Econ. 51, 115–132. doi: 10.1108/IJSE-01-2023-0065

[ref9] Baharuddin T. Nurmandi A. Qodir Z. Jubba H. (2022). Bibliometric Analysis of Socio-Political Research on Capital Relocation: Examining Contributions to the Case of Indonesia. J. Local Govern. Issues 5, 17–31. doi: 10.22219/logos.v5i1.19468

[ref10] Brandtstädter S. (2013). Counterpolitics of Liberation in Contemporary China: Corruption, Law, and Popular Religion. Ethnos 78, 328–351. doi: 10.1080/00141844.2012.688757

[ref130] Chaney P. Sahoo S. (2020). Civil society and the contemporary threat to religious freedom in Bangladesh. J. Civil Society 16, 191–215.

[ref11] de Oliveira Leite R. Dias R. Mendes L. (2021). Morality and Perception of Corruption. Lat. Am. Bus. Rev. 22, 163–188. doi: 10.1080/10978526.2020.1777558

[ref12] Dusha E. (2019). Persistent Inequality, Corruption, and Factor Productivity. He B.E. J. Macroecon. 19:20180021. doi: 10.1515/bejm-2018-0021

[ref13] Etemadinia M. Jahromi A. G. (2022). Religious Interpretations of Near-Death Experiences Based on the Teachings of Shia Islam. Religious Inquiries 11, 171–187. doi: 10.22034/ri.2022.328372.1581

[ref14] Fhima F. Nouira R. Sekkat K. (2023). How does corruption affect sustainable development? A threshold non-linear analysis. Econ. Anal. Policy 78, 505–523. doi: 10.1016/j.eap.2023.03.020

[ref15] Fisher H. E. (2015). The Chinese Inquisition: Xi Jinping’ s War on Corruption 375. Available at:https://egrove.olemiss.edu/hon_thesis/375

[ref16] Frisdiantara C. Indawati N. Wekke I. S. (2017). Religiosity, competence and independence in forming the anti-corruption attitude. J. Eng. Appl. Sci. 12, 1701–1704. doi: 10.3923/jeasci.2017.1701.1704

[ref17] Giorgi A. (2022). Hijack or release? On the heuristic limits of the frame of instrumentalization of religion for discussing the entanglements of populism, religion, and gender. Identities 29, 483–499. doi: 10.1080/1070289X.2021.2002583

[ref18] Gokcekus O. Ekici T. (2020). Religion, Religiosity, and Corruption. Rev. Relig. Res. 62, 563–581. doi: 10.1007/s13644-020-00421-2

[ref19] Gouda M. Park S.-M. (2015). Religious loyalty and acceptance of corruption. Jahrbucher Fur Nationalokonomie Und Statistik 235, 184–206. doi: 10.1515/jbnst-2015-0206

[ref20] Gutterman E. (2023). “Extraterritoriality in the global governance of corruption: Legal and political perspectives” in Research Handbook on Extraterritoriality in International Law. Eds. A. Parrish and C. Ryngaert (Cheltenham, UK: Edward Elgar Publishing Ltd.), 430–442.

[ref21] Habibov N. Afandi E. Cheung A. (2017). Sand or grease? Corruption-institutional trust nexus in post-Soviet countries. J. Eurasian Stud. 8, 172–184. doi: 10.1016/j.euras.2017.05.001

[ref22] Hafner-Burton E. M. Schneider C. J. (2019). Donor Rules or Donors Rule? International Institutions and Political Corruption. AJIL Unbound 113, 346–350. doi: 10.1017/aju.2019.62, PMID: 39801677

[ref23] Han J. (2023). Examining Determinants of Corruption at the Individual Level in South Asia. Economies 11, 179–204. doi: 10.3390/economies11070179

[ref24] Haw T. J. Kueh J. Brahmana R. K. Wei Y. S. (2023). Impact of economic development on corruption in ASEAN countries: panel non-linear model. Int. J. Trade Global Markets 18, 29–49. doi: 10.1504/IJTGM.2023.134919

[ref25] Heath A. F. Richards L. De Graaf N. D. (2016). Explaining Corruption in the Developed World: The Potential of Sociological Approaches. Annu. Rev. Sociol. 42, 51–79. doi: 10.1146/annurev-soc-081715-074213

[ref26] Jha C. K. (2023). Condoning corruption: Who votes for corrupt political parties? J. Econ. Behav. Organ. 215, 74–88. doi: 10.1016/j.jebo.2023.08.026

[ref27] Kang S. Kim D. Kim G. (2023). Corporate entertainment expenses and corruption in public procurement. J. Asian Econ. 84:101554. doi: 10.1016/j.asieco.2022.101554

[ref28] Karim A. Fathurrohman O. Muhammadun Saripudin W. Rahmat D. Mansir F. (2023). Altruistic works, religion, and corruption: Kiais’ leadership to shape anti-corruption values in pesantren. Cogent Soc. Sci. 9:23311886. doi: 10.1080/23311886.2023.2238968, PMID: 39845729

[ref29] Keegan K. (2024). Corruption and digital authoritarianism: political drivers of e-government adoption in Central Asia. Democratization 31, 23–46. doi: 10.1080/13510347.2023.2255146

[ref290] Kidd I. J. (2023). Corrupted temporalities, ‘cultures of speed’, and the possibility of collegiality. Educ. Philosophy and Theory 55, 330–342.

[ref30] Kidd I. J. (2024). Epistemic Corruption and Non-Ideal Epistemology. Int. J. Philos. Stud. 1–7, 1–7. doi: 10.1080/09672559.2024.2418610, PMID: 39845729

[ref31] Kumar M. Sharma A. (2023). Corruption, education and media: review of the film Dasvi (2022). Media Asia 50, 317–323. doi: 10.1080/01296612.2022.2133373

[ref32] Lang M. K. (2014). The Patterns of Corruption in Christian Churches of Cameroon: The Case of the Presbyterian Church in Cameroon. Transformation 31, 132–144. doi: 10.1177/0265378813519724

[ref33] LeClair M. S. (2019). Reported Instances of Nonprofit Corruption: Do Donors Respond to Scandals in the Charitable Sector? Corp. Reput. Rev. 22, 39–47. doi: 10.1057/s41299-018-0056-5

[ref34] Loring N. (2022). “Corruption in Comparison: Clientelism, Nepotism, and Coups D’état in Southeast Asian Politics” in Scandal and Corruption in Congress. ed. Pomante M. J. (Leeds, West Yorkshire, England: Emerald Publishing Limited), 239–260.

[ref35] Maciel G. G. de Sousa L. (2018). Legal Corruption and Dissatisfaction with Democracy in the European Union. Soc. Indic. Res. 140, 653–674. doi: 10.1007/s11205-017-1779-x

[ref36] Malik I. Prianto A. L. Roni N. I. Yama A. Baharuddin T. (2023). “Multi-level Governance and Digitalization in Climate Change: A Bibliometric Analysis” in International Conference on Digital Technologies and Applications. eds. Motahhir S. Bossoufi B. (Cham: Springer), 95–104.

[ref37] Manase Kudzai Chiweshe I. M. (2021). Corruption and the Morality of Everyday Life in Urban Harare. Zimbabwe. Africa Today 68, 73–92. doi: 10.2979/africatoday.68.1.04

[ref38] Marcela Porporato J. I. R. (2016). Changes in government procurement: COVID-19 as an opportunity for corruption Marcela. The B.E. J. Macroecon. 13, 714–735. doi: 10.1108/JAEE-10-2021-0325

[ref39] Marchetti R. Righetti N. Pagiotti S. Stanziano A. (2022). Right-Wing Populism and Political Instrumentalization of Religion. J. Religion Europe 10, 1–28. doi: 10.1163/18748929-bja10052

[ref40] Marshall K. (2008). Ancient and contemporary wisdom and practice on governance as religious leaders engage in international development1. J. Glob. Ethics 4, 217–229. doi: 10.1080/17449620802496347

[ref41] Maswandi J. Zulyadi R. Kartika A. Siregar F. Y. D. (2022). The Role of Islamic Law and Tradition in the Prevention of Corruption by Political Experts in Indonesia. Int. J. Crim. Justice Sci. 17, 114–127. doi: 10.5281/zenodo.4756114

[ref42] Meiryani M. Fernando E. Dewiyanti S. Angelus M. Haliyanti I. (2021). The Effect of Covid-19 on Regional Revenue of DKI Jakarta Province, Indonesia. Proceedings of the 2021 2nd International Conference on Internet and E-Business, 122–128

[ref43] Mensah Y. M. (2014). An Analysis of the Effect of Culture and Religion on Perceived Corruption in a Global Context. J. Bus. Ethics 121, 255–282. doi: 10.1007/s10551-013-1696-0

[ref44] Meričková B. M. Bašteková A. Stejskal J. Pekár B. (2017). Economic, Political, Social Factor of Corruption in the Slovak Republic. NISPAcee J. Public Admini. Policy 10, 99–120. doi: 10.1515/nispa-2017-0005

[ref45] Modesto J. G. Pilati R. (2020). Why are the Corrupt, Corrupt?: The Multilevel Analytical Model of Corruption. Span. J. Psychol. 23, e5–e13. doi: 10.1017/SJP.2020.5, PMID: 32460921

[ref46] Mohammed N. F. Lokman N. Mohamed N. Abu Bakar N. (2023). Exploring anti-corruption education in Malaysian educational institutions. J. Money Laund. Control 27, 284–299. doi: 10.1108/JMLC-02-2023-0037, PMID: 35579975

[ref47] Montes G. C. Luna P. H. (2021). Fiscal transparency, legal system and perception of the control on corruption: empirical evidence from panel data. Empir. Econ. 60, 2005–2037. doi: 10.1007/s00181-020-01849-9

[ref48] Moya Díaz E. Paillama Raimán D. (2022). Sentido, redes y prácticas. Percepciones de la corrupción en gobiernos locales de la Macrozona Sur (Chile). Revista Iberoamericana de Estudios Municipales. 25, 1–24. doi: 10.32457/riem25.1580

[ref49] Nicholson A. Rose R. Bobak M. (2009). Association between attendance at religious services and self-reported health in 22 European countries. Soc. Sci. Med. 69, 519–528. doi: 10.1016/j.socscimed.2009.06.024, PMID: 19595492

[ref50] North C. M. Orman W. H. Gwin C. R. (2013). Religion, corruption, and the rule of law. J. Money Credit Bank. 45, 757–779. doi: 10.1111/jmcb.12024

[ref51] Novella-García C. Cloquell-Lozano A. (2021). The ethics of maxima and minima combined with social justice as a form of public corruption prevention. Crime Law Soc. Chang. 75, 281–295. doi: 10.1007/s10611-020-09921-2

[ref52] Oluseye O. (2024). Exploring potential political corruption in large-scale infrastructure projects in Nigeria. Project Leader. Soc. 5:100108. doi: 10.1016/j.plas.2023.100108

[ref53] Othman W. A. (2023). Christian-Muslim Relations in Early Islam: The Elements and Foundations of their Coexistence. Islam. Q. 67, 65–96.

[ref54] Paldam M. (2001). Corruption and religion adding to the economic model. Kyklos 54, 383–413. doi: 10.1111/1467-6435.00160

[ref55] Portilla J. G. (2022). “Culture, Religion, and Corruption/Prosperity (A), (B), (C), (1), (2)” in Contributions to Economics (pp. 133–183) (Deutschland GmbH: Springer Science and Business Media).

[ref56] Radu I. Sabau M. Sendroiu C. (2015). Coercive economic diplomacy – Corruption trigger or deterrent Economic Computation and Economic Cybernetics Studies and Research/Academy of Economic Studies 49. https://www.scopus.com/record/display.uri?eid=2-s2.0-84944518495&origin=resultslist&sort=plf-f&src=s&sot=b&sdt=b&s=TITLE-ABS-KEY%28Coercive+economic+diplomacy+%E2%80%93+Corruption+trigger+or+deterrent%29&sessionSearchId=68c151902b678525cf800b94c8a48550&relpos=0.

[ref57] Rahbarqazi M. Mahmoudoghli R. (2020). Corruption perceptions, political distrust, and the weakening of political islam in Iraq. Revista Espanola de Sociologia 29, 57–74. doi: 10.22325/FES/RES.2020.57, PMID: 39900720

[ref58] Rontos K. Salvati L. Vavouras I. (2013). Corruption in the world: Its economic, political and geographic determinants and their interactions. J. Reg. Socio-Econ. 3, 5–26.

[ref59] Setia G. Rasyid F. A. Hidayat A. (2023). Political Opposition in Islamic Political Perspective. Russian Law J. 11, 43–52. doi: 10.52783/rlj.v11i3s.735

[ref60] Shi Y. Pan M. (2018). Dynamics of social tolerance on corruption: An economic interaction perspective. Romanian J. Econ. Forecast. 21, 135–141.

[ref61] Singh S. (2005). Fighting Corruption in Developing Countries: Dimensions of the Problem in India. PIARC.

[ref62] Singh D. (2015). Explaining varieties of corruption in the Afghan justice sector. J. Interv. Statebuild. 9, 231–255. doi: 10.1080/17502977.2015.1033093

[ref63] Singh D. (2016). Anti-corruption Strategies in Afghanistan: An Alternative Approach. J. Dev. Soc. 32, 44–72. doi: 10.1177/0169796X15609714

[ref64] Sommer U. Bloompazit P. B. N. Arikan G. (2013). Does faith limit immorality? The politics of religion and corruption. Democratization 20, 287–309. doi: 10.1080/13510347.2011.650914

[ref65] Søreide T. (2016). Corruption and Criminal Justice: Bridging Economic and Legal Perspectives. Cheltenham, UK: Edward Elgar Publishing.

[ref66] Sullins P. (2003). A little knowledge of the Catholic Church. Society 40, 20–24. doi: 10.1007/s12115-003-1031-y

[ref67] Sumaktoyo N. G. Muhtadi B. (2022). Can Religion Save Corrupt Politicians? Evidence from Indonesia. Int. J. Public Opinion Res. 34, 1–12. doi: 10.1093/ijpor/edab029

[ref68] Suyadi S. Hastuti D. Saputro A. D. (2020). Early childhood education teachers’ perception of the integration of anti-corruption education into islamic religious education in bawean island Indonesia. Element. Educ. Online 19, 1703–1714. doi: 10.17051/ilkonline.2020.734838

[ref69] Tahamtan I. Bornmann L. (2019). What Do Citation Counts Measure? An Updated Review of Studies on Citations in Scientific Documents Published between 2006 and 2018. Scientometrics 121, 1635–1684. doi: 10.1007/s11192-019-03243-4

[ref70] Tawiah V. Zakari A. Alvarado R. (2024). “Effect of corruption on green growth” in Environment, Development and Sustainability, vol. 26 (Netherlands: Springer), 10429–10459.

[ref71] White A. (2006). The Paradox of Corruption as Antithesis to Economic Development: Does Corruption Undermine Economic Development in Indonesia and China, and Why Are the Experiences Different in Each Country? Asian-Pacific Law Policy J. 8:1.

[ref72] Wijaya Y. (2014). Constructing an Anti-Corruption Theology. Exchange 43, 221–236. doi: 10.1163/1572543X-12341325

[ref73] Xu X. Li Y. Liu X. Gan W. (2017). Does religion matter to corruption? Evidence from China. China Econ. Rev. 42, 34–49. doi: 10.1016/j.chieco.2016.11.005

[ref74] Zaloznaya M. (2014). The social psychology of corruption: Why it does not exist and why it should. Sociol. Compass 8, 187–202. doi: 10.1111/soc4.12120

[ref75] Zaloznaya M. Gerber T. P. (2021). Social Issues in Contemporary Russia: Women’s Rights, Corruption, and Immigration through Three Sociological Lenses. Annu. Rev. Sociol. 47, 567–586. doi: 10.1146/annurev-soc-090420-100607

[ref76] Zelekha Y. Avnimelech G. (2023). Cultural and personal channels between religion, religiosity, and corruption. Heliyon 9:e16882. doi: 10.1016/j.heliyon.2023.e16882, PMID: 37484323 PMC10360949

